# Single-cell and bulk RNA sequencing-based screening and identification of extracellular trap network-related genes in neutrophils in acute myocardial infarction

**DOI:** 10.1097/MD.0000000000040590

**Published:** 2024-11-22

**Authors:** Wei Li, Jun Yang

**Affiliations:** a The Second Clinical Medical College of Bin Zhou Medical College, Shandong, China; b Yantai Yuhuangding Hospital, Shandong, China.

**Keywords:** acute myocardial infarction, Mendelian randomization, neutrophil extracellular trap, protein–protein interaction network, single cell, weighted gene co-expression network

## Abstract

**Background:**

The neutrophil-mediated generation of neutrophil extracellular traps (NETs) results in an augmented inflammatory response and cellular tissue injury during acute myocardial infarction (AMI). Through the analysis of public database information, we discovered and confirmed putative critical genes involved in NETs-mediated AMI.

**Methods:**

The AMI dataset GSE66360 and the single-cell dataset GSE163465 were downloaded from the Gene Expression Omnibus database. Key genes were screened by bioinformatics. Quantitative real-time PCR (qRT-PCR) was used to verify the key genes, and then a Mendelian randomization (MR) study was conducted on the basis of the genome-wide association study to determine the causal relationship between key genes and AMI. Dimensionality reduction clustering, pseudo-time series, and cell communication were performed on the single-cell dataset to analyze the key genes screened by bulk RNA sequencing and the dynamic evolution of NETs in the AMI process. Immunohistochemistry and Western blot were used to verify the key genes

**Results:**

Six key genes, IL1β, S100A12, TLR2, CXCL1, CXCL2, and CCL4, were screened out through bioinformatics. qRT-PCR results showed that compared with the control group, the expression of 5 key genes was upregulated in the AMI group. In the MR study, CXCL1 and CCL4 were observed to have a causal relationship with AMI. Single-cell analysis showed that NETs-related genes CCL4, CXCL2, and IL1β were highly expressed. Combining single cells, qRT-PCR and MR, gene CCL4 was selected as the focus of the study. H9c2 cardiomyocytes simulated myocardial infarction under hypoxia, and the results showed that the expression of gene CCL4 was increased. The immunohistochemical results of gene CCL4 showed that the expression was upregulated in the AMI group.

**Conclusions:**

We found 6 key genes related to NETs-mediated cell damage during AMI. The results of MR showed that CXCL1 and CCL4 were causally related to AMI. Combining single cells, qRT-PCR and MR, gene CCL4 may play an important role in the AMI process. Our results may provide some insights into neutrophil-mediated cell damage during AMI.

## 1. Introduction

Acute myocardial infarction (AMI) is a dangerous and unstable disease caused by the erosion and rupture of coronary atherosclerotic plaques, followed by the formation of blood clots that block the arteries.^[1]^ Inflammatory responses significantly contribute to the progression of both acute and chronic cardiovascular diseases. The advancement of bioinformatics and diverse technologies has facilitated the identification of various inflammatory markers and prognostic models, which are crucial for predicting the onset of cardiovascular disease. Implantable cardioverter-defibrillators are crucial in preventing sudden cardiac death, heart failure, and myocardial infarction. Inflammation markers may offer insights into predicting the therapeutic efficacy of these devices.^[2,3]^ Albumin is significant in inflammatory processes and influences cardiovascular diseases, including acute coronary syndromes and arrhythmias.^[4,5]^ AMI is one of the most common cardiovascular diseases. Early, rapid, and accurate diagnosis of AMI is the key to initiating effective evidence-based medical management and treatment. Although significant progress has been made in the prevention and treatment of AMI in recent years, the prevalence of AMI has not decreased, but has instead shown an upward trend due to the prevalence of unhealthy lifestyles and the accelerated aging of the population.^[6,7]^ Neutrophil extracellular traps (NETs) are special structures formed after neutrophil necrosis or apoptosis. They are mainly composed of DNA, histones, neutrophil granzymes, lysozymes, antimicrobial peptides, and other molecules. They are important bactericidal weapons of neutrophils and are related to the pathophysiology of various diseases.^[8]^ Since the discovery of NETs, research on them in the cardiovascular field has developed rapidly. Linking NETs with cardiovascular diseases has opened up a new path for the study of pathophysiological mechanisms. Current research results have shown that NETs play an important role in heart failure and myocardial ischemia-reperfusion injury.^[9,10]^ In AMI patients, NETs affect the occurrence and development of the disease by participating in inflammation, oxidative stress, and other mechanisms in the formation of atherosclerotic plaques.^[11]^ However, the role and mechanism of NETs in AMI have not yet been fully elucidated, and the feasibility of NETs in the treatment of AMI has not yet been fully confirmed. Therefore, in-depth and comprehensive research on the regulatory genes and mechanisms related to NETs will help us further understand the physiological significance and potential diagnostic and therapeutic applications of NETs.

## 2. Materials and methods

### 2.1. Data collection

We downloaded the AMI-related dataset GSE66360 and single-cell dataset GSE163465 from the Gene Expression Omnibus (GEO) database. The dataset GSE66360 contains AMI patients (n = 49) and healthy people (n = 50). The single-cell dataset GSE163465 contains the expression profiles of Cd45+ cells isolated from sham-operated (control group) and left anterior descending artery ligated mice 3, 7, and 9 days after AMI.

### 2.2. Screening of differentially expressed genes and construction of weighted gene co-expression networks

First, the GSE66360 dataset was preprocessed, batch corrected and normalized, and differential analysis was performed according to the “limma” package. The absolute value of logFC >1 and the adjusted *P*-value <.05 were used as the screening criteria for differentially expressed genes, and volcano plots and Heatmaps were used for visualization. Weighted gene co-expression network analysis (WGCNA) was used to identify co-expressed gene modules, explore the association between gene modules and AMI, and identify genes within the modules. WGCNA was implemented using the “WGCNA” package. According to the correlation between different modules and AMI, genes with KME values >0.8 in modules with statistically significant correlation were selected as centers.

### 2.3. GO and KEGG enrichment analysis and construction of protein–protein interaction network

The “cluster Profiler” package was used to perform Gene Ontology (GO) and Kyoto Encyclopedia of Genes and Genomes (KEGG) enrichment analysis to help understand the potential pathogenesis of the disease. The STRING website and Cytoscape 3.9.0 software were used to perform protein–protein interaction network (PPI) analysis to identify key genes in the network.

### 2.4. GSEA analysis and nomogram model construction

At the same time, Gene Set Enrichment Analysis (GSEA) was used to divide the genes into high expression group and low expression group according to whether the expression value of the gene was greater than the median. Then the genes were sorted and their differential expression levels were used to check whether all genes were enriched in the pathway. Enrichment at the top indicates that the gene and the pathway are positively correlated, and vice versa. We used the “rms” software package to construct a nomogram model to predict the risk of AMI. Then, we used the “ROC” package to construct a receiver operating characteristic (ROC) curve to evaluate the predictive value of the candidate key genes, and we used the area under the ROC curve (area under the curve, AUC) to represent the discrimination.

### 2.5. Immune cell infiltration analysis

The mobilization and activation of immune cells are involved in the AMI process. Therefore, we used 2 algorithms, Cell-type Identification by Estimating Relative Subsets of RNA Transcripts (CIBERSORT) and single-sample gene set enrichment analysis (ssGSEA), to perform immune infiltration analysis on the gene expression dataset GSE66360 to determine whether activated neutrophils are associated with different disease states and to evaluate the correlation between key genes and immune cells.

### 2.6. Quantitative real-time PCR verification of key genes

We collected peripheral blood samples from 31 patients with acute myocardial infarction and 25 healthy individuals. Total RNA was extracted by lysis with TRIzol lysis buffer, extracted with chloroform, precipitated with isopropanol, and washed with 75% ethanol. The purity and concentration of the obtained RNA were tested, the dilution volume was calculated, reverse transcribed, and quantitative real-time PCR (qRT-PCR) was performed. The data were analyzed using the 2^-(∆∆Ct) method and normalized to GAPDH. Detailed information on all primer sequences is listed in Table S1, Supplemental Digital Content, http://links.lww.com/MD/N988.

### 2.7. Mendelian randomization

Mendelian randomization (MR) is a statistical technique that uses genetic variation as an instrumental variable to explore the causal relationship between exposure and outcome. Single nucleotide polymorphisms (SNPs) were used as instrumental variables to perform MR analysis to explore the causal relationship between key genes and the likelihood of AMI. Genetic data were obtained from publicly accessible genome-wide association study data repositories. The exposure and outcome data for gene CCL4 were ebi-a-GCST90000449 and ukb-a-533, respectively, and the exposure and outcome data for gene CXCL1 were ebi-a-GCST90000458 and ebi-a-GCST90038610. In the MR analysis, inverse-variance weighting was used to assess the association between the levels of key genes and the likelihood of AMI. The study also used the MR-Egger method for additional sensitivity analysis.

### 2.8. Preprocessing of scRNA-seq analysis

To elucidate the function of NETs and pivotal genes in AMI, GSE163465 was employed as the single-cell RNA sequencing (scRNA-seq) analysis dataset, which encompasses the expression profiles of Cd45+ cells extracted from sham-operated (control) and left anterior descending ligated mice at 3-, 7-, and 9-days post-AMI. Initially, quality control was conducted on the raw count data of the dataset. We excluded cells with feature counts exceeding 5000 or below 200, as well as those with a mitochondrial fraction surpassing 20%. The selected data were subsequently normalized. Subsequently, 3000 highly variable genes among the cells were found. The normalized values were scaled to eliminate variance. Third, principal component analysis was conducted on the normalized data. We identify the top 15 significant principal components for optimal dimensional cell-type annotation, which is subsequently utilized for distributional stochastic neighbor embedding (t-SNE) and cell clustering. We choose the initial 15 significant principal components as the best dimensions for the following t-distributed stochastic neighbor embedding (t-SNE).

### 2.9. Cell-type annotation and NETs expression among groups, pseudo-time analysis, and cell communication

The cells were clustered using the “Find Clusters” function. The RunTSNE function was used to operate t-SNE. In order to understand the dynamic changes of immune cells and NETs in AMI, we analyzed the number of each cell population and the expression of NETs in the control group, AMI 3 days, AMI 7 days, and AMI 14 days. The expression of NETs was presented by 4 scoring methods: AUCell, ssGSEA, GSVA, and Add module. In addition, we performed pseudo-time analysis on the expression of neutrophils and NETs, using previously normalized data as input to reveal the differentiation trajectories of neutrophils and NETs. The inferred trajectory was then projected onto a two-dimensional UMAP graph. Next, we divided NETs into 2 groups with high and low expression to understand their expression patterns with different cells and between different groups. Finally, we understood the correlation between different ligand receptors and NETs.

### 2.10. RNA sequencing to screen the expression of key genes

To further understand the expression of key genes screened by bulk RNA sequencing, we visualized the gene expression changes of 6 key genes from the start to the end of the pseudo-time course.

### 2.11. Immunohistochemistry

The AMI mouse model was constructed by ligating the left anterior descending artery. The isolated mouse heart was then immediately placed in a paraformaldehyde solution, fixed with 4% paraformaldehyde for 12 hours, dehydrated with gradient alcohol, transparentized with xylene, embedded in paraffin, and sliced (4–5 μm). Dewaxing and hydration, antigen retrieval, circle drawing with a water-blocking pen, serum blocking, and then incubated with CCL4 receptor antibody in a 4 °C refrigerator overnight. Then, the anti-rabbit/mouse EnVisionTM +/horseradish peroxidase reagent was incubated at 37 °C for 1 hour, and then detected using a DAB staining kit. ImageJ quantitative analysis data

### 2.12. Western blot (WB)

H9c2 cells were utilized to create an in vitro myocardial infarction cell model. These cells were cultivated in Dulbecco’s modified eagle medium supplemented with 10% fetal bovine serum and incubated at 37 °C in a constant temperature incubator following cell resuscitation during the logarithmic growth phase. H9c2 cells were introduced into serum-free culture medium and segregated into 2 groups: 1 group was incubated in a hypoxic environment for 6 hours, while the other group was maintained in a standard incubator. Cells were harvested at the conclusion of the hypoxia incubation period. Cells were lysed to prepare protein samples, followed by gel electrophoresis, blocking, incubation with primary and secondary antibodies, and visualization.

### 2.13. Statistical analysis

PPI analysis was performed according to the STRING website and Cytoscape 3.9.0 software. Statistical analysis and visualization between groups were performed using Graph pad prism9.41. *P* < .05 indicated statistical significance. The rest of the statistical analyses were performed using R4.3.1.

### 2.14. Ethical statement

This study Approval by Ethics Committee of Yantai Yuhuangding Hospital, Approval NO.: 2024-563.

## 3. Results

### 3.1. Screening of AMI differentially expressed genes, construction of WGCNA network, and identification of AMI-related modules

**Figure 1.**
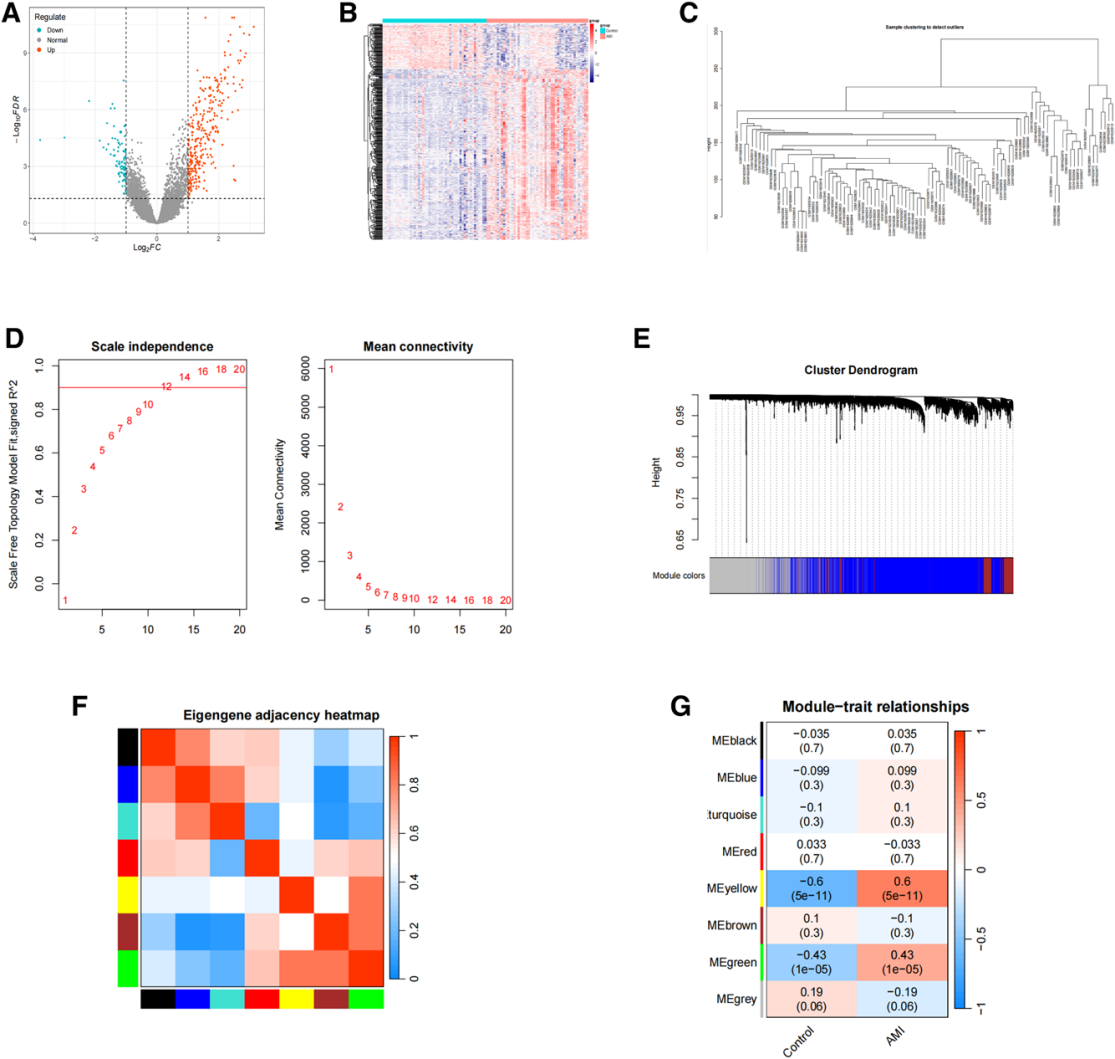
Differential analysis and development of a WGCNA network to uncover gene modules associated with AMI. (A) Red dots in the volcano figure signify upregulated differential genes, blue dots denote down-regulated differential genes, and gray dots indicate genes with no significant differences. (B) Heatmap illustrating the alterations in expression between acute myocardial infarction samples and control samples. Red signifies upregulated differentially expressed genes (DEGs), while blue denotes down-regulated DEGs. The color gradient signifies the magnitude of |log FC| (FC: fold change). (C) Sample clustering for outlier detection. (D) Analysis of mean connection and scale-free indicators over various soft threshold powers. (E) Clustering utilizing the topological overlap matrix (1-TOM) dendrogram of all genes in the GSE66360 dataset. Each branch in the clustered tree signifies a gene, with co-expression modules delineated in various hues. (F) Heatmap illustrating the correlation of each block. (G) Heatmap illustrating the connection between clustered gene modules and myocardial infarction in the GSE66360 dataset. Each module includes the relevant correlation coefficient and *P*-value. AMI = acute myocardial infarction, WGCNA = weighted gene co-expression network analysis.

The GSE66360 dataset was differentially analyzed, and a total of 405 differentially expressed genes were screened, including 310 upregulated genes and 95 down-regulated genes. The comparison between the AMI group and the normal group in the GSE66360 dataset is shown in Figure 1A and B. In order to determine whether the potential gene module is related to AMI, WGCNA analysis was performed on all candidate genes in the AMI-related dataset. We first eliminated abnormal samples by setting a threshold (Fig. 1C). Then the optimal soft threshold was set to 12, as shown in Figure 1D. The clustering tree shows 8 modules of gene co-expression (Fig. 1E). The module correlation Heatmap is shown in Figure 1F. The genes are divided into 8 modules, of which the yellow and green modules have statistically significant correlations with AMI. The number of genes in the 2 modules is 292 and 172, respectively, as shown in Figure 1G. Gene screening was performed according to KME > 0.8. There were 81 genes in the yellow module and 82 genes in the green module. A total of 163 AMI-related genes were screened out from the 2 modules.

### 3.2. GO and KEGG analysis and PPI network analysis of key genes

**Figure 2.**
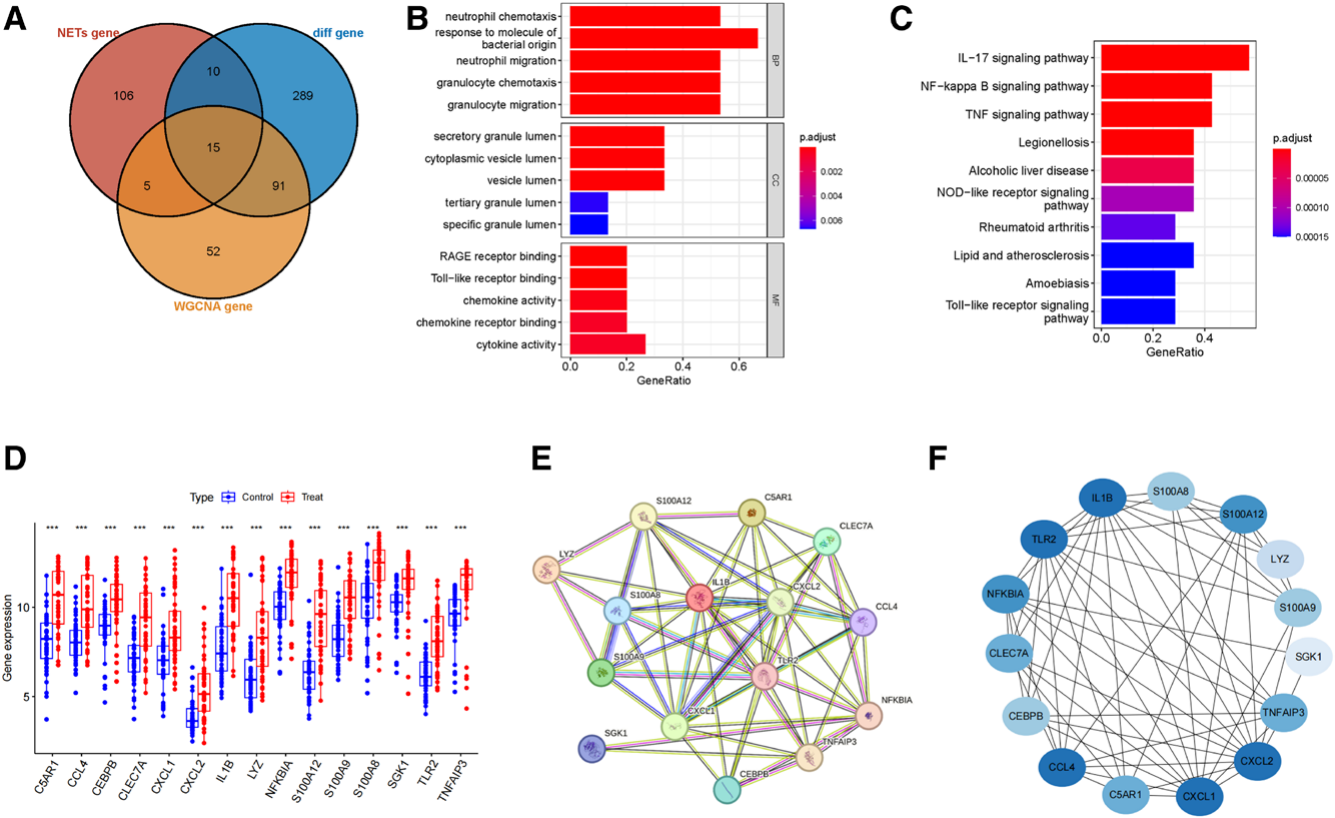
Screening of candidate key genes and construction of the protein–protein interaction (PPI) network. (A) Venn diagram showing 15 intersecting genes. (B) Candidate key genes GO enrichment analysis. (C) Candidate key genes KEGG pathway analysis. (D) Representation of intersecting genes in control and myocardial infarction cohorts. (E) PPI network of intersecting genes. (F) Principal genes of the interoperability network derived from the degree algorithm. GO = Gene Ontology, KEGG = Kyoto Encyclopedia of Genes and Genomes.

The differentially expressed genes, co-expressed network genes, and NETs-related genes were intersected and a Venn diagram was made. There were 15 intersection genes in total, as shown in Figure 2A. These genes are: CCL4, CEBPB, CLEC7A, CXCL1, CXCL2, IL1β, NFKBIA, NLRP3, S100A12, S100A8, S100A9, SGK1, C5AR1, TNFAIP3, and LYZ. These potential intersection genes may have an impact on the occurrence and development of AMI. We used GO and KEGG analysis methods to further study the role of these 15 intersection genes (Fig. 2B and C). KEGG enrichment analysis showed that the intersection genes mainly affected the IL-17 signaling pathway, NF-kappaB signaling pathway, etc. GO enrichment analysis showed that the gene ontology showed that in the cell component category, the intersection genes mainly affected neutrophil chemotaxis, response to bacterial-derived molecules, neutrophil migration, etc. In biological processes, intersection genes are mainly enriched in secretory granule cavities, cytoplasmic vesicle cavities, vesicle cavities, tertiary granule cavities, specific granule cavities, etc. In terms of molecular function, intersection genes are mainly enriched in chemokine activity, chemokine receptor binding, cytokine activity, etc. The expression levels of intersection genes in the control group and AMI are shown in Figure 2D. The intersection genes were subjected to PPI network analysis, and the results showed a total of 15 protein nodes and 62 connecting lines (Fig. 2E). Genes were sequenced according to the CytoHubba plug-in of the Cytoscape software. The darker the color, the higher the sequencing degree of the gene, as shown in Figure 2F.

### 3.3. GSEA analysis and construction and validation of NETs-related nomogram model

**Figure 3.**
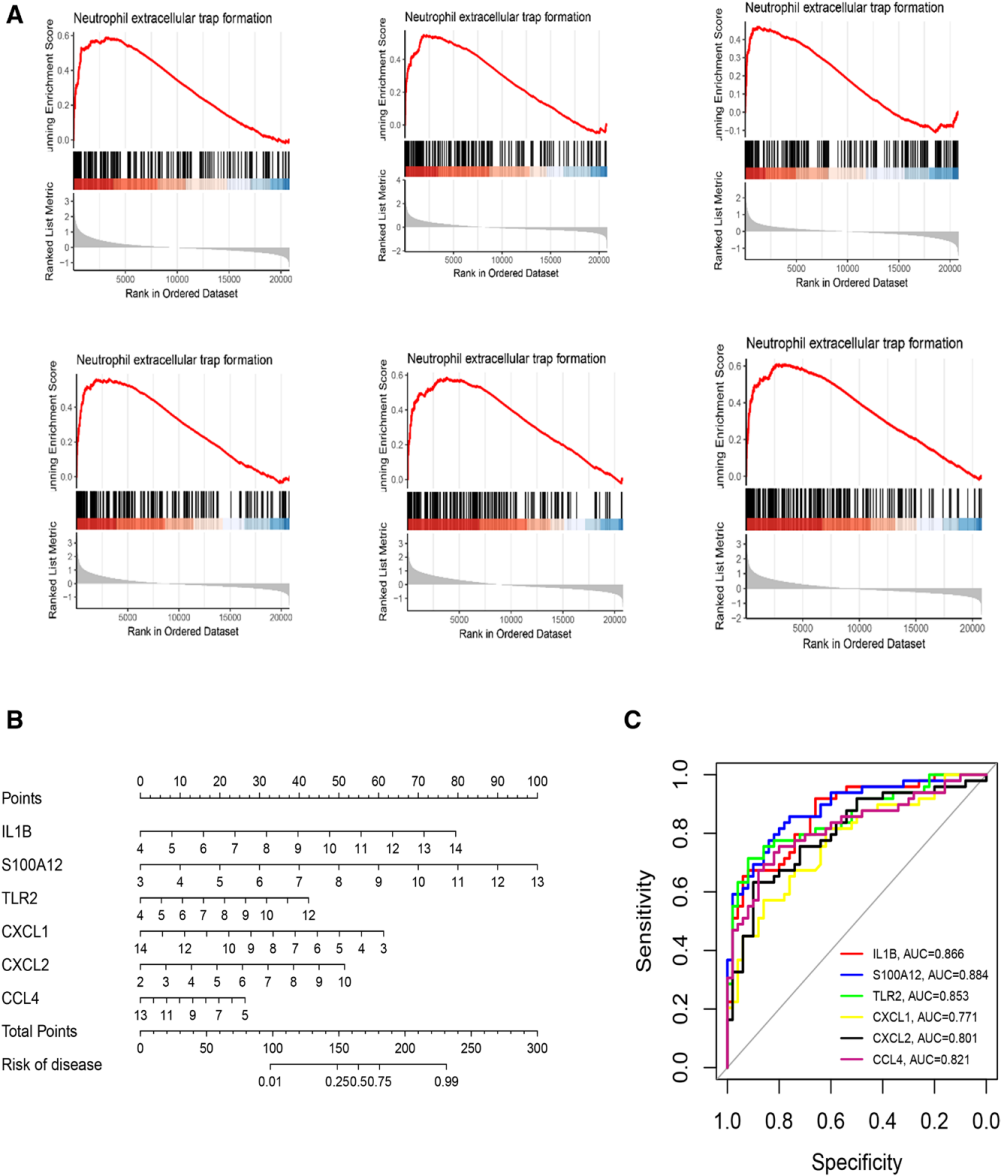
GSEA enrichment analysis of key gene and Column Line Chart Predicts Risk of AMI. (A) Nomogram model of essential genes. (B) ROC curves for assessing the diagnostic efficacy of the nomogram model and each important gene. AMI = acute myocardial infarction, GSEA = Gene Set Enrichment Analysis, ROC = receiver operating characteristic.

We selected the 6 genes with the highest correlation (IL1β, S100A12, TLR2, CXCL1, CXCL2, and CCL4) as potential key genes. We performed gene set enrichment analysis on the 6 key genes in the GSE66360 dataset. The results showed that in the GSE66360 dataset, IL1β, S100A12, TLR2, CXCL1, CXCL2, and CCL4 were all enriched in the NETs formation pathway, and these 6 key genes were positively correlated with the formation of NETs (Fig. 3A). Then a NETs-related nomogram model was constructed to predict the risk of AMI (Fig. 3B). The results showed that our nomogram model performed well in predicting AMI. Subsequently, we calculated the ROC curves of the 6 key genes (IL1β, S100A12, TLR2, CXCL1, CXCL2, and CCL4) to evaluate the diagnostic effect. The model in this study can distinguish AMI from the control group (Fig. 3C). An AUC >0.7 indicates good discrimination, and the results show that the 6 key genes have a good effect in predicting AMI.

### 3.4. Infiltration of immune cells

**Figure 4.**
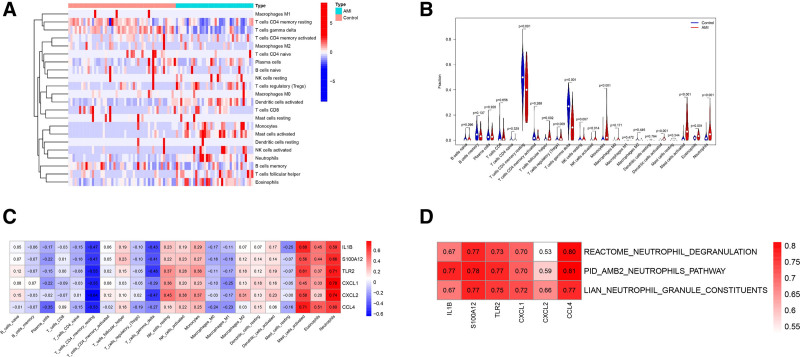
Analysis of immune cell infiltration and correlation analysis of key genes. (A) Heatmap depicting immune infiltration in the AMI and control groups the gradient variation in immune cell coloration signifies| log FC| (FC: fold change) values. (B) Correlation matrix of 22 immune cell subtype constituents. Red: positive correlation; white: congruent correlation; blue: negative correlation. (C) Correlation map of 6 major genes in the GSE66360 dataset with the CIBERSORT algorithm about invading immune cells. (D) Correlation of the 6 principal genes in the GSE66360 dataset utilizing the ssGSEA method with biological pathways linked to NETs creation. AMI = acute myocardial infarction, CIBERSORT = Cell-type Identification by Estimating Relative Subsets of RNA Transcripts, NETs = neutrophil extracellular traps, ssGSEA = single-sample gene set enrichment analysis.

We analyzed the immune cell infiltration of GSE66360 using the ssGSEA algorithm, which compared the expression of 22 immune cell subtypes between the AMI group and the control group. First, the immune cell composition of the samples in the AMI group and the control group was visualized (Fig. 4A), and a violin plot was generated to show the difference in immune infiltrating cells between the AMI group and the control group (Fig. 4B). The violin plot analysis of the difference in immune cell infiltration showed that neutrophils were highly expressed in the AMI group (*P* < .01), which was statistically significant. Then the “CIBERSORT” algorithm was used to evaluate the correlation between key genes and 22 immune cells. The results showed that the 6 key genes were significantly positively correlated with neutrophils, and CXCL1 had the highest correlation with neutrophils (*R* = 0.78) (Fig. 4C). The ssGSEA algorithm was used to evaluate the correlation between the 6 key genes and the biological pathways related to NETs formation. The results showed that they were significantly positively correlated with the biological pathways related to NETs formation, among which the key gene CCL4 had the highest correlation (*R* = 0.81), as shown in Figure 4D.

### 3.5. Identification of key genes by qRT-PCR

**Figure 5.**
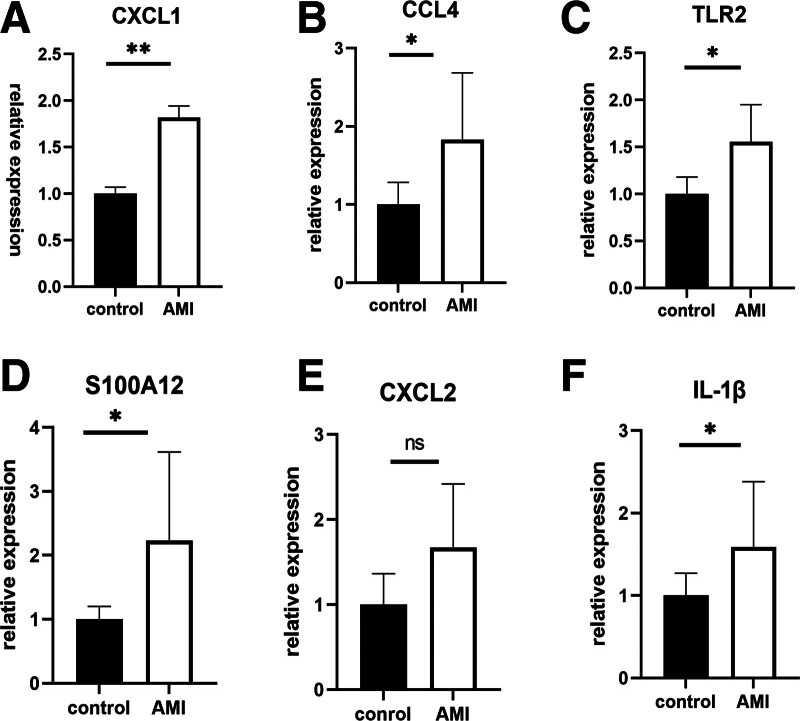
qRT-PCR validation of key genes. (A) Relative expression of CXCL1. (B) Relative expression of CCL4. (C) Relative expression of TLR2. (D) Relative expression of S100A12. (E) Relative expression of CXCL2. (F) Relative expression of IL1β. qRT-PCR = quantitative real-time PCR.

To further confirm the expression patterns of key genes, qRT-PCR validation was performed in blood samples collected from healthy individuals and myocardial infarction groups. The results showed that L1β, S100A12, TLR2, CXCL1, and CCL4 were highly expressed in myocardial infarction samples, consistent with previous analysis, while the expression level of the gene CXCL2 had no significant difference between the 2 groups, which may be due to our insufficient sample size, the result is shown in Figure 5A–F.

### 3.6. CXCL1 and CCL4 are causally related to the risk of AMI

**Figure 6.**
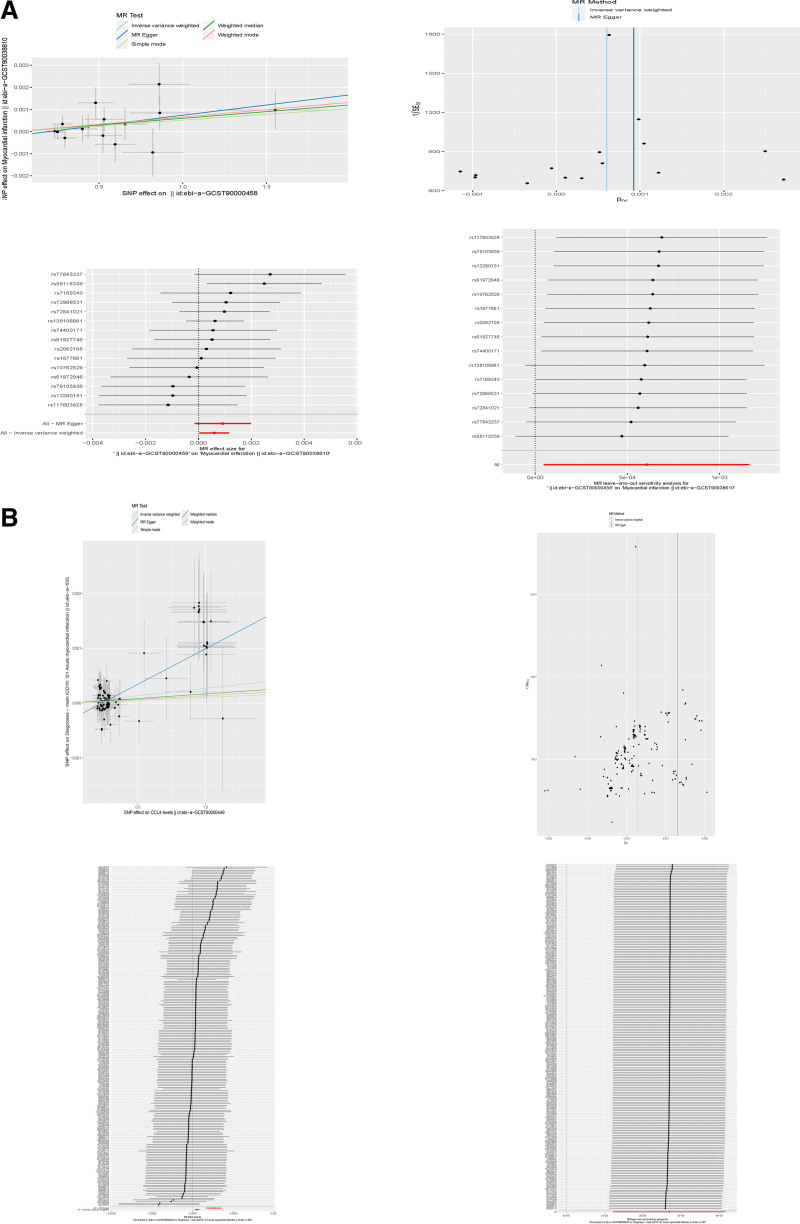
Mendelian randomization. (A) Results of Mendelian randomization studies of CXCL1. (B) Results of Mendelian randomization studies of CCL4.

We selected CXCL1, the gene most related to neutrophils, and CCL4, the gene most related to the formation of biological pathways related to the formation of NETs, to conduct MR analysis to determine the causal relationship between CXCL1 and CCL4 levels and AMI. The results are shown in Figure 6A and B. The scatter plot shows that genes CXCL1 and CCL4 are positively correlated with outcome. The funnel plot of CXCL1 shows an almost symmetrical causal effect. The results of gene CXCL1 using the inverse-variance weighting method show that OR = 1.001 (95% CI = 1.000–1.001, *P* = .03), the MR-Egger method has no statistical significance OR = 1.004 (95% CI = 0.998–1.001, *P* = .11). The funnel plot of CCL4 showed a nearly symmetrical causal effect. The OR value of gene CCL4 = 1.0001 (1.0001–1.0004, *P* = .0003). The MR-Egger method was statistically significant, OR = 1.001 (95% CI = 1.0001–1.001, *P* = 3.93E-07), after excluding each SNP, the MR analysis was repeated for the remaining SNPs. The results consistently showed that all SNPs calculated had significant causal effects, which also indicated that there was no impact on CXCL1 and CCL4 levels and AMI SNPs, thus confirming the validity of the previous MR analysis results.

### 3.7. ScRNA-seq analysis of the dynamics of neutrophils and NETs during AMI

**Figure 7.**
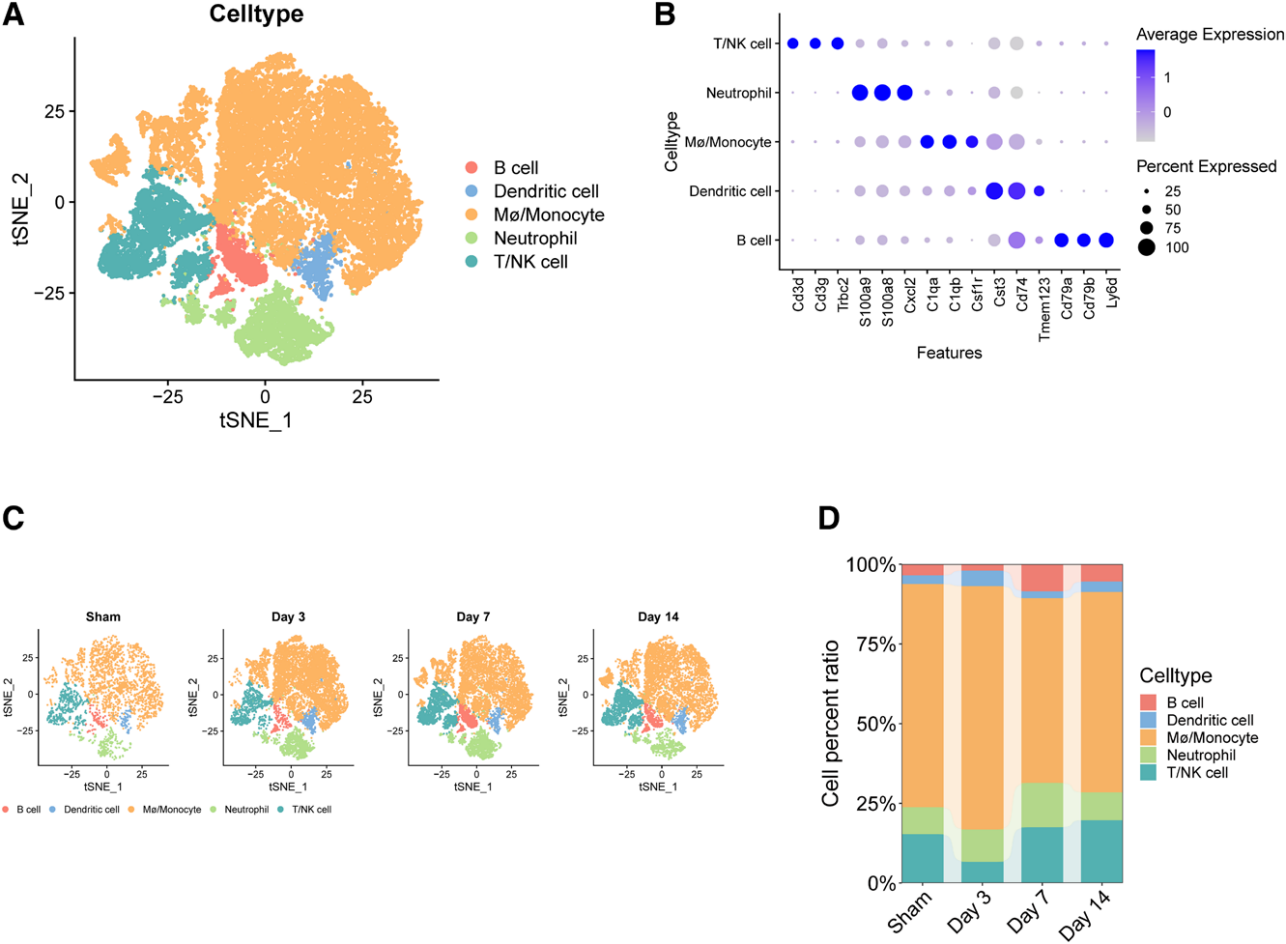
Cell population annotation as well as dynamic evolution. (A) Cell population t-SNE visualization. (B) Cell population marker genes. (C) Cell population expression at different times. (D) Cell population proportion of cells at different times.

To further understand the role of key genes, we used the single-cell dataset GSE16346. Through quality control, unsupervised clustering and t-SNE dimensionality reduction, we identified 5 cell populations. Subsequently, established cardiac immune cell marker genes were used to annotate these 5 putative clusters. Figure 7A depicts the single-cell profiles of these 5 clusters, including neutrophils, macrophages/monocytes, T/NK cells, B cells, and dendritic cells. Figure 7B shows the marker genes of different cell populations: (1) neutrophils, with high expression of Cxcl2, S100a9, and S100a8; (2) macrophages/monocytes, marked by high expression of Csf1r, C1qa, and C1qb; (3) T/NK cells, identified by high expression of Cd3d, Trbc2, and Cd3g; (4) B cells, annotated by high expression of Ly6d, Cd79a, and Cd79b; (5) dendritic cells, with high expression of Tmem123, Cd74, and cst3. Figure 7C and D shows the expression levels of these 5 cell populations at different times.

**Figure 8.**
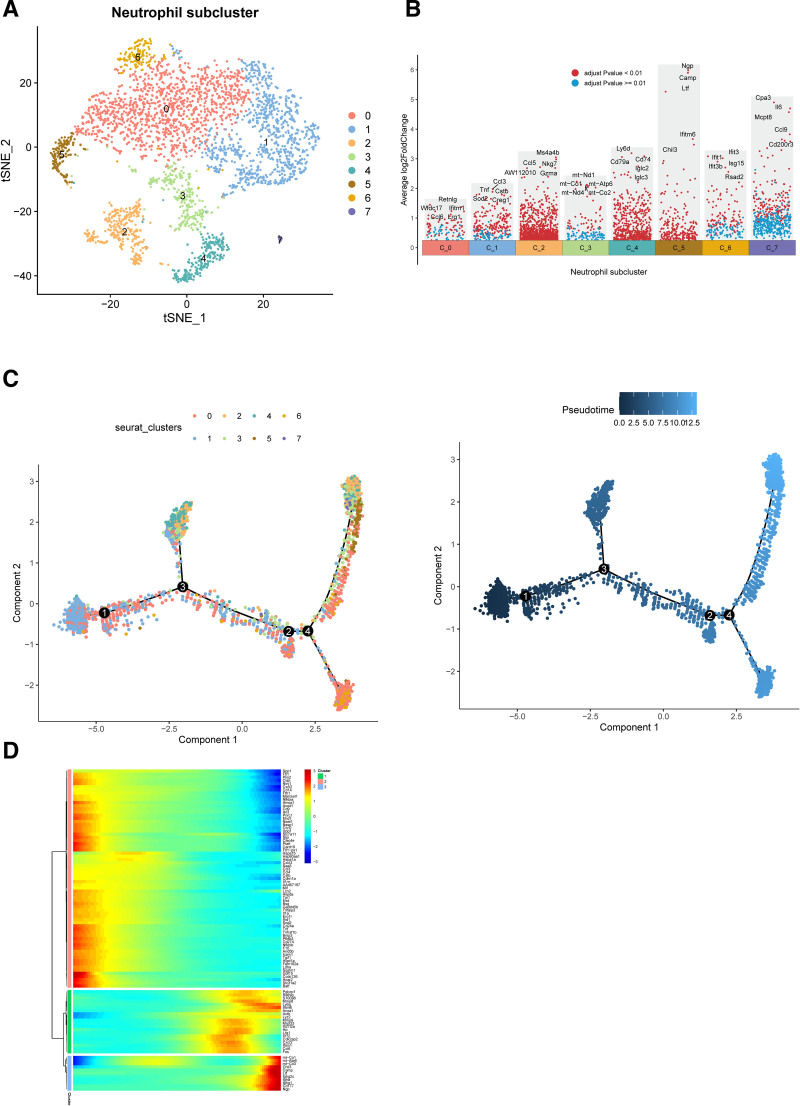
Neutrophil proposed temporal sequence analysis. (A) Annotation of neutrophil subpopulations. (B) Differential genes of subpopulations. (C) Cell track construction. (D) Differential gene analysis.

**Figure 9.**
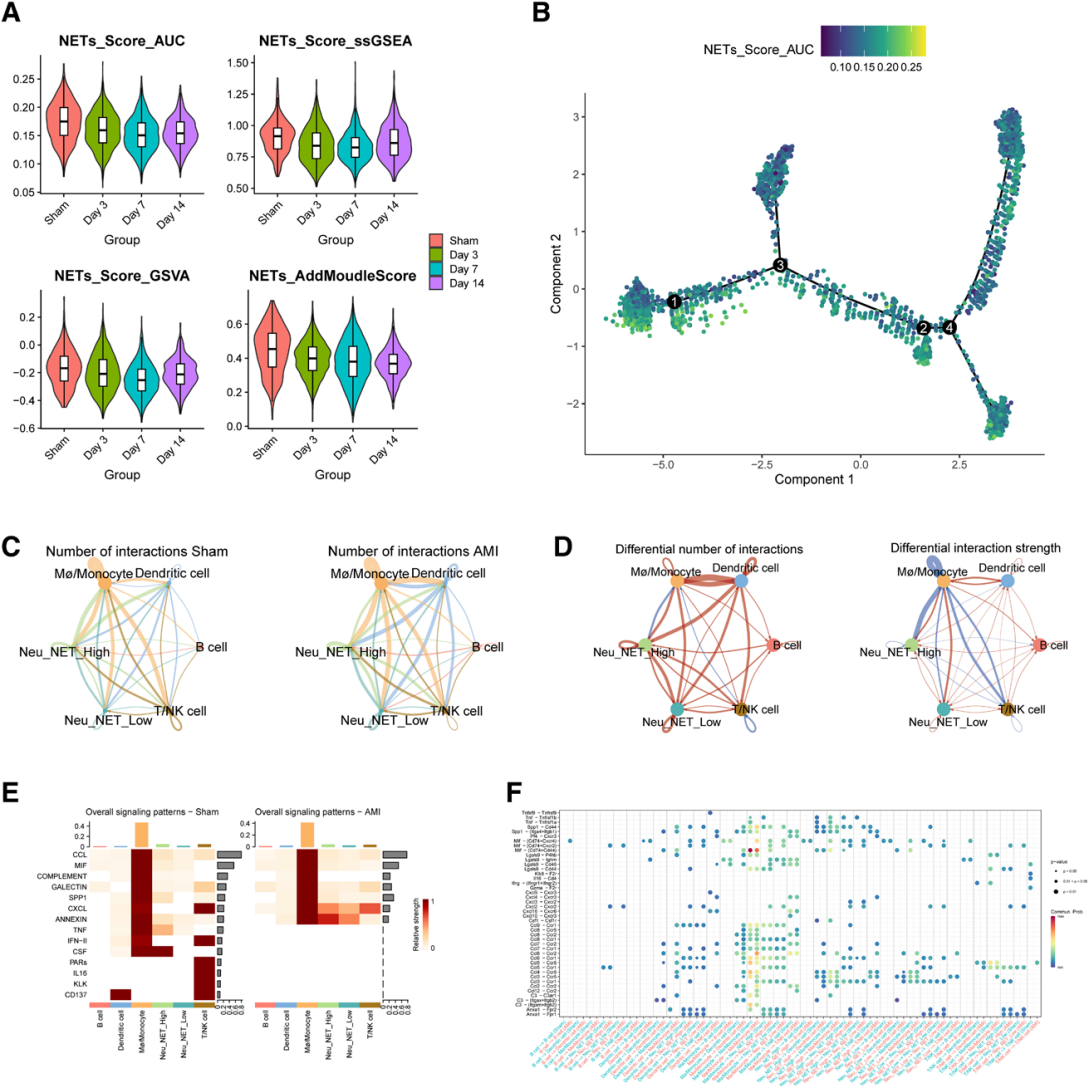
Dynamic evolution of NETs in AMI. (A) Expression of NETs at different times. (B) Proposed temporal trajectory plot of NETs. (C) Analysis of communication between high and low groupings of NETs and different groups of cell populations. (D) Analysis of the strength and probability of interactions between high and low groupings of NETs and cell populations. (E) Analysis of the network of cell populations and pathways. (F) Analysis of ligands and receptors of different cell population. AMI = acute myocardial infarction, NETs = neutrophil extracellular traps.

**Figure 10.**
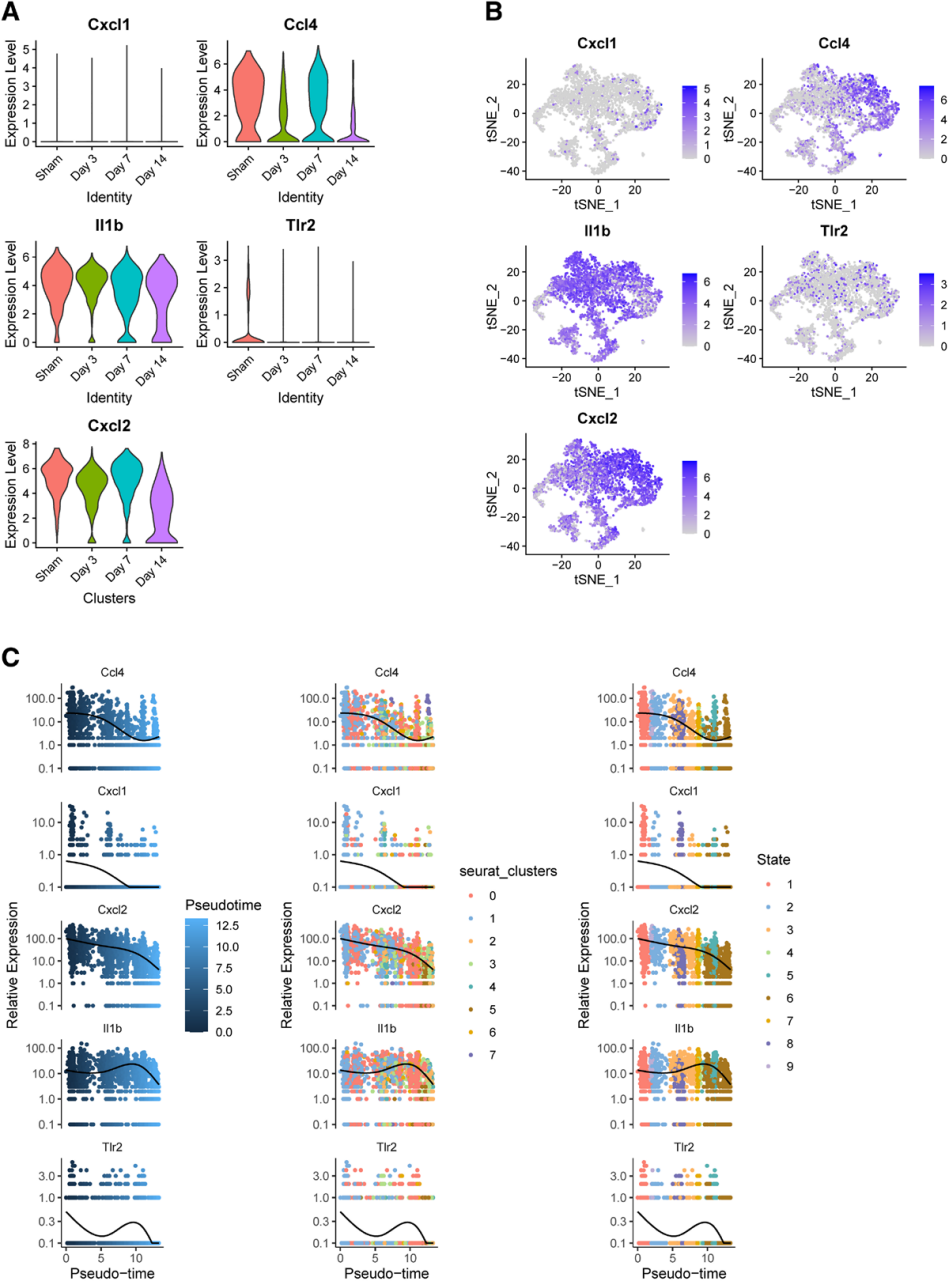
Designated genes visualization. (A) Violin plot of the expression of the designated genes at different times. (B) t-distributed stochastic neighbor embedding (t-SNE) visualization of the designated genes. (C) Proposed chronological analysis of the designated genes.

Neutrophils increased on the 3rd day after myocardial infarction and decreased on the 7th day after myocardial infarction, which may reflect the dynamic evolution of neutrophils during AMI. Then we further reduced the dimension of neutrophils and clustered them, and finally sorted out 8 subgroups (Fig. 8A). The marker genes of each subgroup are shown in Figure 8B. Then, through pseudo-sequential analysis and differential genes of each subgroup, it was shown that the starting point of differentiation may be N1 and N0, and the end point of differentiation may be N4 and N6 (Fig. 8C), which may indicate the process from proinflammatory to anti-inflammatory in the evolution of AMI. The differential analysis divided the expression pattern of TOP100 genes into 3 groups, and the x-axis may indicate the order of AMI evolution (Fig. 8D). Then, through 4 scoring methods, it was shown that the expression of NETs gradually decreased, which may be because inflammatory stimulation is usually most obvious in the acute stage, so the inflammatory activity of neutrophils is higher on days 0 and 3. The gene set score of NETs will also be higher. By 7 and 14 days, the inflammatory response may be weakened, so the gene set score of NETs may decrease, as shown in Figure 9A. The pseudo-time analysis of NETs also showed this (Fig. 9B). The correlation between high and low expression of NETs and other immune cells and ligand receptors was then studied. The results showed that NETs had the greatest correlation with macrophages/monocytes in the sham group and AMI group (Fig. 9C and D), which may indicate that macrophages/monocytes play an important role in the production of NETs. Figure 9E shows that the high expression group of NETs is most correlated with the CXCL and ANNEXIN pathways, which may indicate that chemokines. Figure 9F shows that the ligands cxcl2-cxcr2 and ccl4-cxcr5 play a role in the connection between macrophages, monocytes, and NETs. Next, we studied the expression of the 6 key genes at different times after AMI. The expression of CXCL1 was low, and the expression of gene S100A12 was not found, while the expression of genes IL1β, CXCL2, and CCL4 was high. Combined with pseudo-sequential analysis, it was shown that with the progress of AMI, these inflammatory factors showed a clear downward trend in the late differentiation stage (Fig. 10A–C). Combined with qRT-PCR, MR, and single cells, we selected gene CCL4 for subsequent research.

### 3.8. Immunohistochemistry and WB results

**Figure 11.**
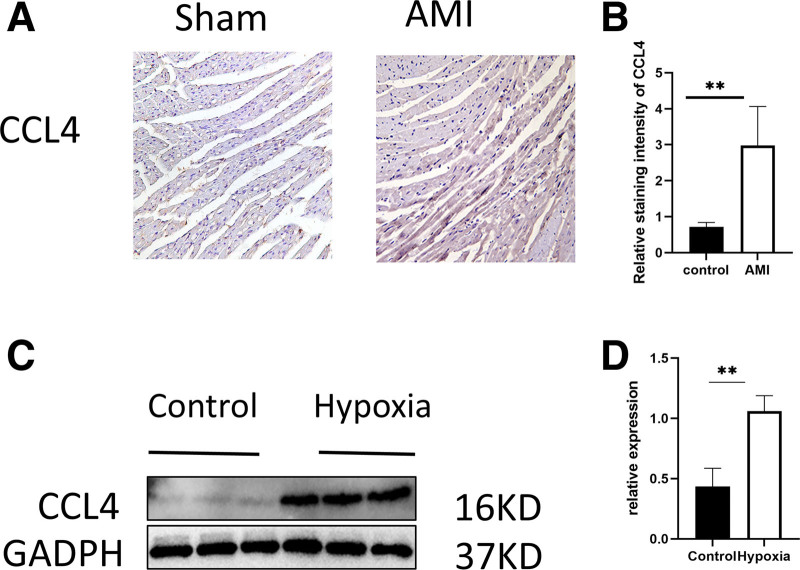
Verification of gene expression. (A) Representative images of immunohistochemical staining of mouse hearts in the myocardial infarction group and the sham-operated group. (B) Statistical analysis of immunohistochemical staining. (C) WB results of H9C2 cells. (D) Statistical analysis of WB. WB = Western blot.

We additionally confirmed the expression of the gene CCL4 via immunohistochemistry, revealing substantial elevation of CCL4 in the infarcted region (Fig. 11A). Statistical analysis revealed a considerable augmentation of positive regions in the infarcted region (Fig. 11B), and WB results suggested that CCL4 was markedly elevated in the injured cell model relative to the control group (Fig. 11C). Statistical analysis indicated a substantial increase in CCL4 expression in the AMI model (Fig. 11D).

## 4. Discussion

AMI is a serious and fatal disease. Multiple risk factors are involved in the occurrence of AMI and affect the prognosis of AMI patients.^[12]^ Megens et al^[13]^ first confirmed and reported the presence of neutrophils and NETs in mouse and human atherosclerotic lesions. Subsequently, Franck et al^[14]^ conducted an in-depth analysis of 56 types of human atherosclerotic plaques. The results showed that NETs are widely present in areas rich in apoptotic endothelial cells and smooth muscle cells, which means that NETs may be involved in plaque rupture, and the distribution of NETs varies according to the type of plaque. Studies have shown^[15]^ that NETs are present in large quantities in all types of complex plaques, and there are no significant differences between plaque types such as intraplaque hemorrhage, erosion, and rupture, but they are not present in intact plaques. Studies have shown that NETs are present in the lumen of atherosclerotic blood vessels and coronary artery specimens of patients after AMI. The accumulation of neutrophils in coronary thrombi^[16]^ and their predictive role in acute coronary events^[17]^ confirm the close relationship between neutrophils and atherosclerotic thrombi. Research results show that coronary thrombi contain a large number of NETs, and NETs are considered to be scaffolds for platelets, red blood cells, and fibrin. Mangold et al^[18]^ found in their study that NETs play a harmful role in the culprit blood vessels of myocardial infarction by stimulating thrombosis and promoting inflammatory responses. In a study of patients with atrial fibrillation,^[19]^ it was also found that NETs may be a risk marker for thrombosis in patients with atrial fibrillation, providing a potential therapeutic target for the management of atrial fibrillation. NETs are released by neutrophils, and in addition to capturing pathogens, they can also mediate the formation of deep vein thrombosis, thereby blocking blood vessels and leading to the development of the disease.^[20]^ The mechanisms involved in NETs have also been reported. NETs are involved in inflammatory responses as well as bacterial killing and clearance. However, their excessive activation can lead to an inflammatory storm in the body, which may damage tissues and cause organ dysfunction.^[21]^ NETs are involved in the activation and dysfunction of multiple overlapping and interacting pathways, including immune responses to injury, inflammation, and coagulation.^[22]^ Using bioinformatics to identify key genes and molecular mechanisms in NETs-related AMI has important clinical significance. Six key NETs-related genes were screened by WGCNA, differential analysis, 3 enrichment methods, and screening. S100 calcium-binding protein A12 (S100A12) is a small calcium-binding protein expressed by neutrophils and monocytes/macrophages, playing an important role in infection, inflammation, and immunity.^[23]^ The NETs-related gene S100A12 plays an important role in the progression of lung adenocarcinoma through the BCYRN1/miR-3664-5p/CTSG regulatory axis.^[24]^ A study showed that plasma S100A12 levels in STEMI patients peaked 1 to 2 hours after symptom onset.^[25]^ Therefore, based on our study and the literature, we speculate that S100A12 may affect the progression of AMI by activating the NETs formation pathway and promoting the formation of NETs. Interleukin 1 beta (IL1β) is a proinflammatory factor. In the subacute phase, IL-1β becomes the main cytokine for cardiomyocyte apoptosis, adverse cardiac remodeling, and heart failure.^[26]^ IL-1β may be a pathogenic factor for neutrophil activation. The main targets of NETs are IL-1β and TNFs.^[27]^ Recent articles have demonstrated that IL-1β is involved in the occurrence of AMI.^[28]^ After acute myocardial infarction, it may activate the release of NETs and release the proinflammatory factor IL-1β. CC motif chemokine ligand 4 (CCL4) is an important factor for the recruitment and activation of leukocytes from the circulation to inflamed tissues. CCL4 is significantly expressed at high levels in neutrophils treated with supernatant derived from breast cancer cells and promotes the formation of NETs.^[29]^ In a mouse model of infarction, CCL4 may drive the recruitment of neutrophils.^[30]^ Combined with our research, CCL4 may participate in the process of AMI by promoting the formation of NETs. Toll-like receptor 2 (TLR2) is a subtype of Toll-like receptor, which is located on the plasma membrane and is one of the important pattern recognition receptors in the immune system.^[31]^ Targeting TLR2 may be a promising strategy to reverse immunosuppression and control tumor progression to improve prognosis.^[32]^ miR-328-3p inhibits the formation of NETs by targeting TLR2, ultimately reducing endothelial cell damage.^[33]^ Inhibiting TLR2 can inhibit inflammation and alleviate thrombosis.^[34]^ GSEA research and database analysis have shown that TLR2 is enriched in the NETs formation pathway and is involved in the formation of AMI. CXC motif chemokine ligand 1 (CXCL1) and CXC motif chemokine ligand 2 (CXCL2) belong to the CXC chemokine family and regulate cardiovascular function under pathological and physiological conditions. Plasma and tissue inflammation are aggravated, which is manifested by increased levels of CXCL1 and CXCL2.^[35]^ The retrograde destruction of endothelial cells by neutrophils is controlled by the enhanced production of chemokines CXCL1 and CXCL2 in mast cells located at the junction of endothelial cells.^[36]^ Our study showed that the genes CXCL1 and CXCL2 are highly correlated with neutrophils and are involved in the formation of NETs. In order to further study the role of key genes screened by bulk RNA sequencing in AMI, we used the single-cell dataset GSE163465 to study the dynamic evolution of key genes and NETs during AMI. The results showed that the expression of NETs and key genes IL-1β, CCL4, and CXCL2 was high in the early stage of AMI, and decreased in the late stage of AMI. This may be because the expression of proinflammatory factors was high in the early stage of inflammation, and their expression decreased during the myocardial cell repair stage. Combining single cells, qRT-PCR, and MR. We selected the gene CCL4 for subsequent research. H9C2 cardiomyocytes simulated the AMI process under hypoxia. Compared with the control group, CCL4 expression increased under hypoxia. Then, immunohistochemistry was used to study the expression of CCL4 in a mouse myocardial infarction model. The results showed that the expression was upregulated compared with the sham group.

Our research comprehensively explores the relationship between the process of NETs and AMI, combines key genes with immune cells and NETs formation for comprehensive analysis, verifies the expression of key genes with qRT-PCR, and explores key gene knots and outcomes through MR analysis causal relationship. And finally identified 6 key genes that may mediate AMI through the formation of NETs, including IL1β, TLR2, CXCL1, CXCL2, CCL4, and CXCL2. MR analysis showed that CXCL1 and CCL4 are causally related to AMI. Single cells research shows that the expression of IL1β, CXCL2, and CCL4 is high. The gene CCL4 was selected for subsequent research based on the 3 parts. The results of WB and immunohistochemistry showed that CCL4 is upregulated during AMI. The limitation of this study is MR. In the study, no suitable exposure data were found for the genes IL1β, TLR2, CXCL2, and CXCL2, and in the qRT-PCR study, there was a significant expression difference between the groups for the gene CXCL2, possibly because of the small number of samples.

## 5. Conclusion

Taken together, this study may provide insights into neutrophil-mediated cellular damage during acute myocardial infarction.

## Author contributions

**Conceptualization:** Jun Yang.

**Writing – review & editing:** Wei Li.

## Supplementary Material


