# Supplementary material for: Single-cell and bulk RNA sequencing-based screening and identification of extracellular trap network-related genes in neutrophils in acute myocardial infarction
Source: Medicine (Baltimore). 2024 Nov 22;103(47):e40590. doi: 10.1097/MD.0000000000040590 (PMC11596368; doi:10.1097/MD.0000000000040590)
Supplement: Supplementary file 1 [file medi-103-e40590-s001.docx]

Table S1 Primer sequences

| Gene name | presequence | subsequence |
| --- | --- | --- |
| CCL4 | GCTAGTAGCTGCCTTCTGCT | CCACAAAGTTGCGAGGAAGC |
| CXCL1 | AGGGAATTCACCCCAAGAACA | ATGCAGGATTGAGGCAAGC |
| TLR2 | CTGTGCTCTGTTCCTGCTGA | GATGTTCCTGCTGGGAGCTT |
| CXCL2 | TTGTCTCAACCCCGCATCG | GTTGGATTTGCCATTTTTCAGC |
| S100A12 | CTCAGTTCGGAAGGGGCATT | TGGTAATGGGCAGCCTTCAG |
| IL1β | CCAGCTACGAATCTCCGACC | TATCCTGTCCCTGGAGGTGG |
| GADPH | GGAGCGAGATCCCTCCAAAAT | GGCTGTTGTCATACTTCCATGG |
